# Caution before canonisation: a clinical and implementation science perspective on psychedelics

**DOI:** 10.3389/fpsyt.2026.1922928

**Published:** 2026-10-05

**Authors:** Andreas Paris, Foteini Thoma, Aikaterini Velentza, Panagiota Pavlopoulou, Giorgos Metaxas

**Affiliations:** 1 Centre for Research in Intellectual and Developmental Disabilities, University of Warwick, Coventry, United Kingdom; 2 Specialized Psychodiagnostic and Evidence-Based Consultation Services, Code Behaviour/Modus Mores, Nicosia, Cyprus

**Keywords:** implementation science, MDMA-assisted therapy, psilocybin, psychedelic-assisted therapy, psychoactive treatment innovation, translational psychiatry

## Abstract

Recent advances in psychedelic research have renewed interest in the potential therapeutic role of psychedelic-assisted interventions in psychiatry. Early clinical trials suggest that compounds such as psilocybin and MDMA, administered within structured therapeutic frameworks, may produce rapid symptom improvements for conditions including depression, post-traumatic stress disorder, and substance use disorders. While these findings are scientifically promising, the current evidence base remains characterized by methodological and translational limitations. Many studies involve small samples, short follow-up periods, functional unblinding, and heterogeneous safety reporting, which complicate causal interpretation and generalizability. In the present paper, we examine psychedelic therapies through clinical, methodological, ethical, and implementation science lenses. We argue that current trials primarily demonstrate proof-of-concept efficacy under controlled conditions rather than readiness for large-scale clinical adoption. Responsible translation therefore requires stronger evidence regarding durability, safety monitoring, comparative effectiveness, and real-world implementation. Psychedelic therapies represent an important emerging research direction, but continued rigorous evaluation and improved implementation of established evidence-based interventions remain central to addressing mental disorders.

## 1 Introduction

Mikellides and Kyriazis (1) have recently argued that psychedelic-assisted therapies may represent a transformative reimagining of psychiatric care, highlighting emerging evidence suggesting rapid symptom reduction, potentially durable benefit, and experiential processes such as catharsis, insight, and compassion. Their contribution reflects a broader resurgence of scientific interest in psychedelic psychiatry and an increasing willingness within the field to explore novel therapeutic paradigms for conditions that remain difficult to treat using existing approaches. At the same time, the empirical foundation supporting such transformative claims remains an evolving evidence base that continues to require careful evaluation against contemporary standards for evidence synthesis, causal inference, and implementation readiness (2–5).

Our perspective also complements recent consensus statements on the integration of psychedelic therapies into routine clinical care, including the US National Network of Depression Centre’s Task Group recommendations for psilocybin (6). Although these recommendations primarily focus on the safe clinical implementation of psilocybin-assisted treatment, the present paper adopts a broader implementation science perspective across psychedelic-assisted interventions.

This perspective paper does not argue against continued psychedelic research. Rather, it seeks to complement the growing optimism within the field by emphasizing the importance of evidentiary proportionality and translational rigor consistent with standards applied to other psychiatric and clinical interventions. Although the present article was prompted in part by recent position papers on psychedelic psychiatry, including Mikellides and Kyriazis (1), the considerations discussed here reflect broader dynamics within the contemporary psychedelic literature. We thus seek to complement that perspective by examining the evidentiary and implementation requirements necessary for responsible clinical translation.

Recent syntheses consistently show that psychedelic literature is characterized by relatively small samples, short follow-up windows, narrowly defined recruitment populations, and persistent methodological challenges, particularly expectancy effects and functional unblinding (2–4). These limitations do not negate the promising signals emerging from early trials, but suggest that the field remains in an early translational phase. As a result, there is a reasonable concern that enthusiasm for psychedelic interventions may, at times, move toward premature canonization before the evidence base can fully substantiate claims regarding exceptional efficacy, long-term durability, scalability, or equity of access (3, 4, 7).

The present perspective therefore evaluates psychedelic-assisted therapies from clinical, ethical, and implementation science perspectives. In doing so, we aim to situate the emerging psychedelic evidence base within the broader realities of psychiatric treatment development and health system translation. Specifically, we consider the current state of clinical evidence, the methodological challenges inherent in psychedelic trials, safety and ethical considerations, and the requirements for responsible large-scale implementation. We also emphasize that mental health systems may achieve substantial benefit by improving the implementation of established evidence-based psychotherapies and medical interventions that already possess robust empirical support but remain under-delivered in routine care (8–11).

## 2 Clinical evidence and methodological limitations

Literature suggests that psychedelic-assisted therapies may produce short-term symptom improvements under highly controlled research conditions and this inference is broadly consistent with findings from several recent systematic reviews and meta-analyses examining psychedelic-assisted therapies across multiple indications, including depression, post-traumatic stress disorder, and substance use disorders (12, 13, 45). Several randomized trials, including psilocybin trials for major depressive disorder and treatment-resistant depression as well as phase-3 MDMA trials for post-traumatic stress disorder, have reported clinically meaningful short-term symptom reductions in carefully controlled settings (14–16).

Recent randomized controlled trials have continued to demonstrate encouraging, although heterogeneous, findings. These include the COMP360 program examining psilocybin for treatment-resistant depression (15, 17), recent phase 2 studies of inhaled 5-MeO-DMT (18), and additional controlled investigations across depressive disorders (19). Collectively, these studies reinforce the therapeutic promise of psychedelic-assisted interventions while simultaneously highlighting persistent variability in response rates, durability, participant selection, and trial methodology.

These findings though, should not yet be interpreted as demonstrating superiority over existing treatments or as establishing a paradigm-shifting therapeutic modality within routine psychiatric care (2–5). Even where effect sizes appear large, meta-analytic syntheses consistently report substantial heterogeneity and reduced certainty once methodological limitations are taken into account (2–4).

A central validity challenge in psychedelic research concerns expectancy effects and functional unblinding. Because the acute psychoactive profile of classic psychedelics is often unmistakable, participants, and sometimes investigators, may be able to infer treatment allocation, thereby increasing the likelihood that expectancy influences reported outcomes (3, 4, 20). In comparative analyses, Metaxa and Clarke (4) demonstrated that psilocybin effect sizes vary meaningfully as a function of outcome measurement strategies and study design features consistent with expectancy amplification, including reliance on self-report outcomes and contextual variables. Similarly, Hsu et al. (3) reported that comparative effectiveness estimates for psilocybin may be inflated when unsuccessful blinding and expectancy bias are not adequately accounted for, and that recalibration against placebo responses in antidepressant trials reduces the effect magnitude.

These considerations are central to the interpretation of psychedelic clinical findings because they complicate causal attribution. Observed improvements may reflect pharmacological effects, expectancy amplification, contextual influences, or interactions among these mechanisms (3, 4). Addressing these interpretive challenges is therefore essential for determining the true magnitude and mechanism of therapeutic benefit. Concerns regarding expectancy effects and narrative amplification are also discussed at the broader field level. Critical syntheses have suggested that early psychedelic findings may be situated within a broader cycle of scientific and media enthusiasm, in which promising early signals receive amplified attention before the evidence base fully matures (21). Such dynamics are not unique to psychedelics. Similar trajectories have been observed in other areas of psychiatric innovation, where initial effect sizes often attenuate as trials become larger, more methodologically rigorous, and more representative of real-world populations.

Another methodological constraint concerns the absence of placebo conditions fully suited to psychedelic trials. Although randomization remains standard practice, the development of credible active placebos that match psychoactive onset, subjective intensity, and physiological effects remains an ongoing challenge (3, 4, 22). Recent methodological guidance has therefore emphasized the importance of developing innovative placebo strategies capable of preserving masking while avoiding therapeutic effects within the target condition (22). Such advances would help address the risk that participants, particularly those with prior psychedelic experience, may identify allocation and inadvertently influence outcome reporting. Independent methodological convenings have similarly highlighted functional unblinding and expectancy structure as unresolved design challenges that must be addressed before strong efficacy claims can be generalized beyond carefully controlled research environments (23).

The recognition of these methodological challenges should not be interpreted as an argument against psychedelic research but rather as a roadmap for improving future trial designs. Several strategies have been proposed to strengthen internal validity, including the development of more credible active placebo conditions, low-dose psychedelic comparator arms, systematic assessment of blinding integrity throughout trials, expectancy-adjusted statistical analyses, and the inclusion of pharmacological comparators capable of producing comparable subjective effects without equivalent therapeutic action (22, 24, 25). Recent methodological guidance has also recommended re-evaluation of recruitment strategies, inclusion criteria, and pragmatic trial designs to improve internal validity and real-world generalizability.

Finally, psychedelic trials often incorporate intensive preparation and integration components, which are clinically reasonable but complicate causal inference. When therapeutic outcomes emerge from a complex interaction between pharmacology, psychological preparation, therapist support, and environmental context, it becomes more difficult to distinguish drug-specific effects from context-mediated meaning-making (26). This dynamic reinforces the importance of study designs capable of separating pharmacodynamic mechanisms from contextual amplification when interpreting psychedelic trial outcomes (3, 26). In this context, framing psychedelics as exceptional pharmacological solutions risks overstating what current trials can conclusively establish (4, 5).

Recent naturalistic and observational research further underscores the importance of contextual and relational variables in shaping psychedelic outcomes. A recent scoping review identified a rapidly expanding body of naturalistic studies while also highlighting considerable heterogeneity in study populations, settings, contextual variables, and reporting practices (27). In another example, a large-scale international survey study (N = 1,867) found that therapeutic-like context and perceived relational support significantly moderated associations between life stress, challenging psychedelic experiences, and subsequent psychological outcomes (28). Importantly, these findings were derived from retrospective self-report data and cross-sectional analyses, limiting causal inference and generalizability. While such work highlights the potential importance of set, setting, and therapeutic support, it also reinforces the extent to which observed outcomes may be driven by contextual, relational mechanisms or broader psychotherapeutic processes, rather than psychedelic-specific effects alone. This further complicates attribution of therapeutic efficacy and underscores the need for controlled designs capable of disentangling drug-specific from context-mediated effects.

Similarly, a recent study compassionate-use cohort, reported significant improvements in depression and anxiety following psychedelic-assisted psychotherapy delivered within routine specialist clinical services (29). While this provides encouraging evidence regarding feasibility under real-world conditions, the retrospective, uncontrolled nature of the study limits causal inference and highlights the importance of high-quality comparative trials.

## 3 Durability of effects

Claims of long-lasting benefit after one or two psychedelic sessions remain an area of active investigation. Current systematic reviews indicate promising short-term outcomes but note that controlled long-term data of sufficient breadth, rigor, and representativeness are still limited (2, 4, 5). Across existing syntheses, follow-up windows are typically relatively short (often 6 to 12 weeks), and longer-term outcomes are frequently inferred from open-label extensions rather than demonstrated in adequately powered maintenance or relapse-prevention designs (2, 5).

At the same time, several emerging studies suggest that rapid antidepressant effects following psychedelic administration may persist for clinically meaningful periods in some individuals. For example, early clinical studies involving both classical psychedelics and shorter-acting psychedelic compounds have reported sustained improvements over weeks to months following single or limited dosing sessions. These findings highlight the potential therapeutic promise of psychedelic interventions while also reinforcing the need for longer-term controlled trials capable of determining durability across broader populations and clinical settings.

Importantly, some of the longest available follow-up data come from studies involving patients with cancer-related psychological distress. For example, long-term follow-up of the original NYU trial demonstrated sustained reductions in anxiety, depression, hopelessness, demoralisation, and death anxiety for up to 4.5 years following psilocybin-assisted psychotherapy (30). Similar recent studies have also reported encouraging improvements in depressive symptoms and psychological well-being among patients with cancer (31). While these findings support the potential durability of psychedelic-assisted interventions, they emerge from highly selected clinical populations receiving intensive psychotherapeutic support and should thus not yet be assumed to generalize across broader psychiatric disorders or routine clinical practice.

Ketamine provides a useful analog when considering this translational challenge. Its rapid antidepressant effects are among the most robust findings in contemporary psychopharmacology. However, these improvements do not consistently translate into sustained remission without maintenance strategies, and relapse frequently occurs within weeks in many samples (55). This observation does not diminish the clinical value of rapid-acting treatments. Rather, it illustrates the importance of distinguishing acute response from sustained recovery and underscores why durability claims for emerging psychedelic approaches require maintenance trials, pragmatic designs, and longer-term follow-up before being framed as transformative therapeutic advances (3, 55).

Ongoing developments within ketamine research further illustrate that the challenge of sustaining early therapeutic gains remains an active area of investigation even for comparatively well-established rapid-acting interventions. A recent randomized controlled trial demonstrated that low-dose buprenorphine administered following ketamine significantly prolonged reductions in suicidal ideation compared to placebo, thus highlighting continued efforts to optimize maintenance strategies after an initial rapid response (32). Rather than diminishing ketamine’s established efficacy, such work illustrates that treatment optimization and durability remain central translational challenges across rapidly acting psychiatric interventions.

Future research should therefore prioritize adequately powered maintenance trials, relapse-prevention studies, and pragmatic effectiveness trials extending beyond the relatively short follow-up periods that characterize much of the current literature. Longer-term observational cohorts including functional recovery, quality of life, and occupational functioning would also provide a more comprehensive understanding of whether early symptom improvements translate into durable clinical benefit under routine practice conditions (3, 55). These approaches would substantially strengthen confidence regarding the long-term therapeutic value of psychedelic-assisted interventions.

## 4 Safety and risk considerations

Safety considerations are equally important as psychedelic research moves toward broader clinical translation. While classic psychedelics are often described as physiologically safe in controlled settings and lack traditional dependence potential, careful evaluation of potential risks remains essential (3, 7). Acute adverse psychological reactions, including panic, dysphoria, paranoia, and transient psychotic-like experiences, have been documented even under supervised research conditions (7, 33). In addition, clinical observations suggest that psychedelic exposure may precipitate manic or psychotic episodes in vulnerable individuals, highlighting the importance of careful screening, longitudinal monitoring, and appropriate post-acute support pathways (34).

Comprehensive contemporary synthesis of adverse events in classic psychedelic studies emphasizes several important limitations in the existing safety literature, including heterogeneity in adverse-event definitions, inconsistent methods of harm ascertainment, limited follow-up durations, and the frequent exclusion of participants at elevated psychiatric risk. Together, these factors constrain the generalizability of safety conclusions and highlight the need for more systematic pharmacovigilance as research expands (7).

Recent systematic reviews continue to indicate that serious adverse events remain relatively uncommon within carefully controlled clinical settings. However, further syntheses consistently identify substantial heterogeneity in adverse-event definitions, inconsistent ascertainment procedures, limited follow-up, and variability in reporting standards, all of which constrain confidence in current safety estimates and reinforce the need for prospective pharmacovigilance as psychedelic therapies move toward broader clinical implementation (5, 7).

Under such conditions, the absence of reported harms in highly selected research samples should not automatically be interpreted as evidence of safety in broader clinical populations or routine care environments (7). This concern is reinforced by broader findings in clinical trial methodology showing that adverse events are frequently underreported unless explicit guideline-concordant reporting standards are used. The updated CONSORT Harms reporting framework provides a useful benchmark for improving transparency and consistency in the reporting of safety outcomes in psychedelic trials (35).

Future studies would similarly benefit from greater standardization of adverse-event definitions, prospective pharmacovigilance frameworks, longer-term surveillance of psychiatric and medical outcomes, and routine adoption of contemporary harms-reporting standards (24). Such developments would facilitate more accurate estimation of both common and uncommon adverse outcomes while improving comparability across studies and informing regulatory decision-making as psychedelic therapies move towards broader clinical evaluation.

Ketamine provides a useful comparison when considering safety evaluation in rapidly evolving treatment fields. Although ketamine demonstrates strong short-term antidepressant effects, repeated exposure has been associated with dissociation, cognitive effects, misuse potential, and urological toxicity, including ketamine-associated cystitis, particularly with sustained or non-medical use (36, 37). These observations illustrate that treatment safety is not limited to acute physiological toxicity but also includes longer-term behavioral, neurological, and systemic effects. Accordingly, responsible clinical translation requires that therapeutic enthusiasm be accompanied by careful safety monitoring and transparent risk communication as interventions move from tightly controlled trials into more heterogeneous real-world populations (7).

## 5 Speculative combinations

Furthermore, proposals to combine psychedelics with repetitive transcranial magnetic stimulation (rTMS) illustrate how emerging therapeutic hypotheses may extend beyond the current evidentiary base. While rTMS is now supported by substantial evidence and contemporary international guidelines as an intervention for treatment-resistant depression, ongoing developments including accelerated protocols like the Stanford Neuromodulation Therapy (SNT/SAINT, 38), and personalized stimulation approaches that continue to refine its clinical effectiveness. Nevertheless, questions remain regarding optimal patient selection, protocol standardization, comparative effectiveness, and long-term durability, particularly as newer stimulation paradigms continue to emerge. Historical.meta-analytic findings showed variable outcomes, with conclusions sensitive to protocol parameters and study quality (39–41). International guidelines therefore recommend rTMS primarily as an adjunctive intervention rather than a broadly generalizable first-line modality, reflecting its indication specificity and the still-evolving nature of its evidence base (41, 56).

From a clinical translation standpoint, combining two interventions, namely psychedelics and rTMS, each characterized by ongoing questions regarding durability, generalizability, and mechanism may increase rather than reduce uncertainty. Such combinations are therefore best considered hypothesis-generating rather than clinically actionable until supported by appropriately designed trials with credible masking, robust harms monitoring, and functional outcomes (3, 7). Psychiatry has seen analogous cycles of early enthusiasm followed by more constrained clinical roles when rigorous evaluation refined initial expectations, including within neuromodulation research where early narrative momentum occasionally preceded durable evidence (42). Thus, rather than pursuing increasingly complex multimodal interventions prematurely, an important research priority may be the optimization of individual treatment components through robust mechanistic investigation before combination approaches are evaluated within adequately powered clinical trial designs (24).

## 6 Psychological processes, subjective experiences and neurobiological uncertainty

Concepts such as catharsis, compassion, and insight are increasingly discussed in psychedelic psychiatry, yet they are rarely operationalized or tested as mechanisms of therapeutic change. These constructs are clinically meaningful and may reflect important aspects of patient experience; however, claims regarding them as reliable mechanisms of symptom remission require careful qualification. Empirical evidence suggests that psychedelics may influence certain psychological and interpersonal processes, yet effects are partial, heterogeneous, and dependent on measurement strategies and contextual variables (43).

Beyond the aforementioned terms, contemporary psychedelic literature has increasingly focused on additional psychological constructs including mystical-type experiences, ego dissolution, increased openness, psychological flexibility, and cognitive flexibility, as potential contributors to therapeutic change (44, 45). These frequently emerge following psychedelic administration and may represent important aspects of patients’ subjective experiences but, considerable uncertainty remains regarding their causal contribution to clinical improvement. While several studies report associations between them and symptom reduction, mediation findings remain heterogeneous, and many proposed mechanisms continue to rely on observational analyses rather than experimental confirmation (21, 44). Thus, these are best regarded as candidate mechanisms requiring further empirical validation rather than established explanations of therapeutic efficacy.

For example, systematic evidence indicates small-to-moderate changes in mindfulness-related constructs (e.g., acceptance and non-reactivity), although results vary widely across designs and populations, limiting mechanistic certainty (43). Similarly, meta-analytic evidence suggests that classic psychedelics may increase emotional empathy while showing less consistent or absent effects on cognitive empathy, which complicates broader claims of generalized prosocial transformation (46).

Neurobiologically, competing explanatory models emphasize 5-HT2A receptor agonism, network-level changes, and neuroplasticity, while other frameworks emphasize subjective “mystical-type” experiences as mediators of therapeutic outcomes. However, current evidence does not yet support definitive mechanistic validation, and there remains a risk of conflating phenomenological descriptions with causal mechanisms (26). Accordingly, constructs such as compassion or catharsis are best understood as promising hypotheses requiring direct empirical testing rather than established scientific explanations of therapeutic action (43).

From a neuropsychological perspective, the dominant outcome architecture in psychedelic trials remains primarily symptom-centric and comparatively limited in its assessment of broader functional outcomes. Symptom scale change does not necessarily map onto improvements in cognition, executive functioning, judgment, or real-world capability, which are domains that ultimately determine occupational functioning, relational stability, and relapse vulnerability (7). This concern is further emphasized by the role of set and setting, which may amplify belief salience and narrative coherence, potentially producing post-acute decisional shifts not captured by conventional symptom measures (26). Accordingly, stronger endpoint frameworks incorporating functional outcomes and capability measures alongside symptom change would help clarify both benefits and potential trade-offs (7, 35).

## 7 Implementation science and evidence-based interventions

An implementation science perspective helps clarify what is currently missing from much of the psychedelic evidence base: demonstrable capacity for equitable, scalable, and sustainable delivery. The Reach, Effectiveness, Adoption, Implementation, and Maintenance (RE-AIM) and Consolidated Framework for Implementation Research (CFIR) frameworks emphasize reach, adoption, fidelity, maintenance, and contextual determinants as prerequisites for real-world impact, not optional considerations following efficacy trials (47, 48). Psychedelic trials to date typically involve narrowly selected samples, intensive therapist involvement, specialized infrastructure, and carefully curated therapeutic environments that may be difficult to replicate at scale. As a result, the current evidence base is best interpreted as proof-of-concept efficacy under ideal research conditions rather than readiness for system-wide translation (3, 4).

Recent analyses examining potential translation of psychedelic therapies within European health systems further emphasize that regulatory approval alone does not guarantee adoption. Health technology assessment frameworks require evidence of comparative effectiveness, cost-effectiveness, workforce feasibility, and added value relative to existing standards of care, which are the exact criteria that psychedelic therapies have not yet consistently met (54). This reinforces the important distinction between experimental promise and implementable clinical innovation.

Implementation science also distinguishes clinical outcomes from implementation outcomes such as acceptability, feasibility, fidelity, penetration, sustainability, and cost. Without measurement of these domains, an intervention’s real-world impact cannot be reliably inferred from efficacy trials alone (49). Process frameworks such as Exploration, Preparation, Implementation, Sustainment (EPIS) similarly emphasize that adoption and sustainment depend on determinants operating at system, organizational, clinician, and patient levels, which must be explicitly operationalized rather than assumed (50). Tools such as the Pragmatic-Explanatory Continuum Indicator Summary-2 (PRECIS-2) framework formalize the explanatory-pragmatic continuum in trial design and help determine whether evidence is suitable for informing adoption decisions; by these criteria, much of the psychedelic literature remains relatively explanatory and therefore limited in generalizability to routine care settings (51).

Against this background, a clinically conservative and ethically defensible priority is to improve implementation of established evidence-based interventions that already demonstrate effectiveness but remain under-delivered in routine care. Evidence-based psychotherapies and established pharmacotherapies have extensive empirical support across diverse populations, yet their delivery is constrained by workforce shortages, training limitations, reimbursement barriers, and inconsistent fidelity monitoring (8–10). Collaborative care and stepped-care models offer scalable pathways for improving mental health outcomes at population level without requiring speculative pharmacological paradigms (9, 10).

From an implementation perspective, future research would benefit from greater use of hybrid effectiveness-implementation designs, multi-center pragmatic trials, formal health-economic evaluations, and systematic measurement of implementation outcomes including feasibility, fidelity, acceptability, sustainability, and equity. These approaches would substantially strengthen the evidence required for responsible health-system integration should regulatory approval continue to expand. Furthermore, over the past several years, increasing attention has been directed toward the systematic assessment of subjective psychedelic experiences using a growing range of psychometric instruments and mediation models. Nevertheless, substantial heterogeneity remains in both the constructs assessed and the measures employed, limiting direct comparison across studies and preventing definitive conclusions regarding whether these experiences constitute causal mechanisms of therapeutic change or represent important correlates of treatment response.

In this sense, the central challenge in global mental health care is less a shortage of innovation than a persistent failure of implementation (10). Until scalable delivery, safety monitoring, and functional benefit beyond highly controlled settings are demonstrated, psychedelic-assisted therapies are best framed as emerging investigational approaches rather than replacements for established medical and psychotherapeutic standards of care (7, 49).

## 8 Ethical considerations

Ethical considerations further underscore the need for careful translation. Psychedelic states can increase suggestibility and emotional lability, complicating informed consent and potentially increasing vulnerability to boundary violations. These characteristics necessitate rigorous consent processes, strong governance structures, and explicit safeguards against misconduct in both research and clinical contexts (52). Furthermore, expectancy inflation and amplified cultural narratives surrounding psychedelic therapies may also distort risk communication and contribute to therapeutic misconception, thereby raising the threshold for conservative claims in both research dissemination and clinical translation (3, 4).

Informed consent represents a particularly important ethical consideration within psychedelic-assisted therapy because acute alterations in perception, emotional processing, and suggestibility may influence participants’ understanding of treatment expectations, potential risks, and therapeutic uncertainty. Contemporary guidance therefore recommends enhanced consent procedures that explicitly address expectancy effects, altered states of consciousness, uncertainty regarding therapeutic mechanisms, and the experimental nature of many psychedelic interventions (53). Such approaches may help minimize therapeutic misconception while promoting genuinely informed patient decision-making.

Moreover, commercialization introduces additional complexities. Therapist-intensive protocols and boutique-clinic delivery models risk concentrating access among affluent populations, potentially widening inequities unless deliberate public-health implementation strategies are incorporated from the outset (9, 10). By contrast, expanding access to established evidence-based psychotherapies and medical approaches through system-level delivery innovations represents a more equitable and immediately actionable strategy for reducing mental health burden at population level (8, 10).

Equity considerations are also central to regulatory decision-making. Within Europe, new pharmacological interventions typically require both European Medicines Agency (EMA) approval for safety and efficacy and health technology assessment evaluation (HTA) to determine whether a treatment provides added clinical value and can be sustainably incorporated into national health systems (54). Such frameworks aim to ensure that emerging treatments are not only scientifically credible but also accessible and economically sustainable. In contrast, accelerated regulatory designations such as “breakthrough therapy” status in the United States may increase public expectations even while evidence continues to develop. Without parallel evaluation of cost-effectiveness and comparative effectiveness, early enthusiasm may inadvertently contribute to inequities in access and availability. These dynamics highlight the importance of transparent scientific communication and ongoing regulatory evaluation as new treatments emerge. While such processes may temper early enthusiasm, they ultimately protect patient safety and preserve equitable access to care.

## 9 Conclusion

The resurgence of psychedelic research represents an important and scientifically valuable development in contemporary psychiatry. Early clinical signals in selected populations warrant continued investigation, and recent studies highlight the potential therapeutic relevance of psychedelic-related interventions. At the same time, the current literature remains constrained by methodological challenges including expectancy effects, functional unblinding, limited follow-up duration, restricted external validity, and inconsistent reporting of harms (2–4, 7). Phenomenological constructs such as catharsis and compassion may represent meaningful aspects of patient experience but cannot substitute for validated mechanisms, durable clinical effectiveness, functional outcomes, and implementation readiness (43, 46).

For readers of Frontiers in Psychiatry, the implication is straightforward. Progress in psychedelic psychiatry will most likely emerge through rigorous research addressing methodological challenges, long-term outcomes, and transparent harms reporting, alongside careful attention to implementation feasibility and health system integration. Psychedelics may ultimately assume a defined role within psychiatric care. However, as with any emerging intervention, that role will need to be established through the same evidentiary standards routinely applied across medicine and supported by implementation science demonstrating feasibility, fidelity, equity, and sustainability at scale (47–49). Until such evidence accumulates, responsible clinical stewardship suggests prioritizing both continued investigation of psychedelic therapies and improved implementation of already evidence-based psychotherapies and established medical approaches (8, 10, 11).
